# Herbal medicine use in adults who experience anxiety: A qualitative exploration

**DOI:** 10.3402/qhw.v10.29275

**Published:** 2015-12-15

**Authors:** Erica McIntyre, Anthony J. Saliba, Carmen C. Moran

**Affiliations:** 1 School of Psychology, Charles Sturt University, Wagga Wagga, Australia; 2 National Wine and Grape Industry Centre, Charles Sturt University, Wagga Wagga, Australia

**Keywords:** Anxiety, beliefs, attitudes, herbal medicine, complementary medicine

## Abstract

Herbal medicine use is widespread and has been reported to be as high as 21% in people with anxiety disorders. Critical thematic analysis was used to explore beliefs and attitudes towards herbal medicines in adults experiencing anxiety. In-depth interviews were conducted with eight adults who experienced anxiety and used herbal medicines. Three major themes were found: *Herbal medicines being different from pharmaceuticals*, *evidence and effectiveness*, and *barriers to herbal medicine use*. Within these themes people held beliefs about the safety of natural treatments, valued anecdotes from friends and family as a form of evidence for self-prescribing, and described confusion about herbal medicines and their cost as barriers to using them as a treatment option. The findings will inform future research and provide guidance for health practitioners.

Herbal medicine is a popular complementary and alternative medicine (CAM) used throughout the world, with lifetime prevalence of use reported as high as 37% in Australia (Thomson, Jones, Evans, & Leslie, 2012). Herbal medicines are used to treat a range of health concerns, including common mental health problems such as anxiety (Bystritsky et al., 2012; Parslow & Jorm, 2004). The prevalence of herbal medicine use has been reported to be as high as 21% in patients with anxiety disorders (Bystritsky et al., 2012). Anxiety is the most prevalent mental health problem in Australia, with a reported 26% of Australians having an anxiety disorder diagnosis in their lifetime (Slade, Johnston, Oakley Browne, Andrews, & Whiteford, 2009). In addition, an unknown number of people experience problematic “sub-threshold” anxiety who do not meet the criteria for a disorder and may not be identified as needing treatment (Grenier et al., 2011; Kessler & Wittchen, 2002).

Evidence-based treatments for anxiety symptoms include both psychological interventions (e.g., cognitive behavioural therapy) and pharmaceutical drugs (e.g., selective serotonin reuptake inhibitors). Although these treatments provide relief for many people, they are not always effective (Huh, Goebert, Takeshita, Lu, & Kang, 2011; Taylor, Abramowitz, & McKay, 2012), may not align with people's beliefs (Prins, Verhaak, Bensing, & Van der Meer, 2008), have unwanted side effects, or are difficult to access (due to cost, location, or stigma) (Baldwin, Woods, Lawson, & Taylor, 2011; Prins et al., 2008). These barriers to conventional treatment may be a factor causing people to consider herbal medicines as an alternative, or as a complement to evidence-based treatments to further relieve symptoms. Other clinical groups (e.g., cancer patients) and general population samples have been found to use herbal medicines because they are dissatisfied with their conventional treatments (Shumay, Maskarinec, Gotay, Heiby, & Kakai, 2002; Sirois & Gick, 2002). However, this usage has yet to be explored in adults experiencing anxiety.

Although there are benefits in having an alternative treatment choice for anxiety symptoms, herbal medicines can be problematic if used incorrectly. For example, people who self-medicate with herbal medicines may place themselves at risk of not receiving the most effective treatments. In addition, there is a risk of people using herbs incorrectly, such as taking the wrong dosage or using less effective preparations (e.g., non-standardized extracts), or choosing herbs that can interact with pharmaceuticals. Herbal medicines are currently being used concurrently with pharmaceuticals. For example, in a general population sample, Zhang, Story, Lin, Vitetta, and Xue (2008) found that 28.8% of participants (*n*=2526) took both a pharmaceutical treatment and a herbal medicine for the same condition in the last 12 months, as they received no benefit from the pharmaceutical. In addition, they found that 51.8% of herbal medicine users (*n=*571) self-prescribed them.

Previous research found that adults with anxiety and other mental health conditions self-prescribe herbal medicines, using them concurrently with pharmaceuticals and not disclosing this use to their healthcare providers (Knaudt, Connor, Weisler, Churchill, & Davidson, 1999) (Alderman & Kiepfer, 2003). For example, in a cohort of psychiatric outpatients (*N=*213)—50% of whom had a diagnosis of an anxiety disorder—CAM users (which included herbal medicines) used these treatments concurrently with pharmaceuticals for their psychiatric (63%) and physical (68%) symptoms (Knaudt et al., 1999). In addition, 49% of CAM users did not disclose their herbal medicine use to their doctors. Another study of adult psychiatry patients (*N*=52) found that 51.9% had used either herbal or vitamin supplements, with 37% of these users not disclosing this use to their doctors (Alderman & Kiepfer, 2003). These studies present a concerning trend of self-prescription of herbal medicines with concurrent use of pharmaceutical medicines that creates a risk of harm.

Research has identified a number of beliefs and attitudes that predict the intention to use herbal medicines in both the general population and specific clinical groups. Predictors identified include specific belief systems (e.g., holism, postmodern values) (Bishop, Yardley, & Lewith, 2006; Siahpush, 1999), treatment beliefs and attitudes (e.g., faith in natural treatments, dissatisfaction with the medical encounter) (O'Callaghan & Jordan, 2003; Siahpush, 1998), and control and empowerment beliefs and attitudes (e.g., rejection of authority and personal control over health) (O'Callaghan & Jordan, 2003; Thomson, Jones, Browne, & Leslie, 2014). However, these characteristics may not reflect the beliefs and attitudes of people who experience anxiety and use herbal medicines.

As some people experiencing anxiety choose to use herbal medicines, potentially using them unsafely, an understanding of the beliefs and attitudes that lead to their intention to use herbal medicines is needed. This insight will contribute to understanding the level of risk in the community so that the relative need for future research can be determined and policy developers can address educational needs or other risk interventions. Although previous studies have provided some insight into the beliefs about and attitudes to herbal medicines in a range of cohorts, no study to date has explored these phenomena in adults experiencing anxiety. Therefore, the aims of this study are to explore the beliefs about and attitudes toward herbal medicines of adults who have experienced anxiety and how these individuals make decisions about herbal medicine use.

## Materials and methods

Ethical approval for the study was provided by the Charles Sturt University Human Ethics Committee and conformed to the Declaration of Helsinki.

### Sample strategy and participants

Purposive sampling was used to recruit Australian adults (18 years of age and over) who had experienced anxiety symptoms and had used herbal medicines. People were recruited through advertisements in health practitioner clinics (general practitioners, herbalists, naturopaths, and psychologists), on Facebook, via university forums and newsletters, and through snowballing. A total of eight people (two males, six females) were interviewed from the Blue Mountains and Central Western areas of New South Wales, Australia. The age range was 37 to 69 years. Most interviewees were employed as professionals, with two being university students of mature age. Recruitment ceased at eight people as no new insights were developing relative to the research objectives (Mason, 2010).

### The role of the researcher

This research was approached using a social constructionist perspective. The ontological assumption was that truth is subjective and constructed by an individual's perceptions, experiences, and social relationships (Andrews, 2012). The epistemology assumes that the researcher has an inevitable role in influencing the interviewee's story, as the interviewer has existing knowledge and experience that will influence a person's responses and interpretation of those responses (Patten, 2002). Because this perspective assumes each person defines their own reality, a predetermined definition of *anxiety* was not used to recruit participants (Andrews, 2012), and the subjective lived experience of each person was acknowledged.

The truthfulness and meaningfulness of the data were ensured through use of an audit trail to document the data collection and analysis to ensure transparency and record decision-making as part of a reflexive process (Burr, 2015). This process was used throughout to assist in identifying potential bias influenced by the interviewer's background as a Western herbalist that may have compromised the perspective of each interviewee. During the interviews it was discovered that three of the participants knew the first author was an herbalist; therefore it was explained to these participants that the focus of the interview was on exploring their own subjective experience. There were no notable differences in the themes identified between the participants who knew the interviewer was an herbalist and those who did not.

### Data collection and analyses

Data collection and analyses occurred concurrently over 4 months between October 2013 and January 2014 and were conducted by the first author. After responding to advertisements, participants were contacted by the first author and given information about the study. A time and place of mutual agreement were then arranged for the interviews. Interviews were conducted at a location that was convenient to the participants. Participants received a $15 grocery voucher to acknowledge their generosity for participating. Prior to beginning the interviews, participants completed a consent form ensuring informed consent. The interview guide incorporated questions from Joos, Glassen, and Musselmann (2012), who explored herbal medicine use in cancer patients, with additional questions developed from reviewing the herbal medicine use literature. Questions covered topics related to decision-making in herbal medicine use and the participant's experiences using herbal medicines (see Appendix A for interview questions). The interview guide was semi-structured, using open-ended questions that were asked of each participant, with different probing questions used as needed to encourage richer responses or added as a result of an iterative reflexive process that identified additional themes needing to be explored. Each interview lasted between 30 and 45 min. Interviews were audio recorded on a computer. The interviewer transcribed (verbatim) audio recordings from each interview.

The analytic purpose of the study was to explore and identify implicit and explicit semantic themes related to the beliefs and attitudes that adults with some experience of anxiety held towards herbal medicines and treatment decision-making, which would be used to inform the development of a questionnaire. A critical thematic analysis was used because it is flexible and applies a clear process to organize and describe data, while allowing for interpretation (Braun & Clarke, 2006). Familiarization with the data began during transcription of each interview. NVivo 10 was used to code and organize the data into themes. Each transcribed interview was read initially and coded. Code descriptions were developed that reflected the entire dataset; they were revised as needed, as transcripts were read and reread. Codes were organized into themes that reflected the analytic objectives of the study. The second author assessed a sample of text for consistency of coding relative to the theme definitions, and there was 100% intercoder agreement. The thematic definitions and categories were revised as links between concepts were developed in relation to the broader meanings and relationships that were identified. To ensure developing themes were grounded in the data, they were checked against the data throughout the analysis. To ensure the anonymity of participants, pseudonyms were used and descriptive data was removed when reporting the results.

## Results

### Description of participants and their herbal medicine use

All participants had a subjective experience of anxiety, with two having been diagnosed with generalised anxiety disorder (i.e., GAD). The remaining six participants described anxiety that was secondary to other problems, such as stress, insomnia, or depression. The anxiety experienced by those without a diagnosis was significant enough for them to seek treatment for their symptoms. All participants described getting relief from anxiety in varying degrees from using herbal medicines; however this did not occur in every instance they were used, as described by “Betty.”

Betty: I have used St John's wort as well for I guess that's more anxiety and depression … that didn't work so well for me.

Participants reported using herbal medicines to treat a range of conditions, such as digestive problems, muscular pain, allergies, glandular fever, depression, insomnia, and anxiety. These medicines were taken in a variety of ways, including as teas, tablets, capsules, liquid formulas, and creams. Participants reported using a range of herbs such as chamomile, valerian, and St John's wort. There was a varied amount of herbal medicine use among participants, with some having tried them only a few times and others taking them as part of their regular healthcare. The way in which herbal medicines were prescribed also varied, with some people self-prescribing only, others only using herbs prescribed by a practitioner, and some using a combination of both approaches.

Sally: I've been prescribed things in the past. Um, but because I've got a little bit of knowledge about things, and I know where to go for information I do tend to self-prescribe.

Participants’ first experiences with herbal medicine use ranged from having used as a child through to recent use as an adult. All participants had some positive attitudes towards herbal medicines, although within their interviews some people contradicted these attitudes and remained cautious about using them.

All participants said they would be willing to disclose their herbal medicine use to a health practitioner. However, in most cases they did not see why disclosure was necessary unless it was directly related to the issue being treated. While most people stated they did not care what their doctor thought about their herbal medicine use, some participants contradicted this as they described avoiding discussing it for fear of not being taken seriously. For example, when asked if he tells his doctor he uses herbal medicines, David replied, “I do say I've had the odd vitamin C and those sort of things. But the other things I don't … I think my current doctor's rather sceptical about all those things … yeah just some of the things he says, well I think, you know, I'm not going to mention that.”

### Major themes

Three major themes were identified that reflected beliefs and attitudes to herbal medicines and treatment decision-making in adults having experienced anxiety: 1) herbal medicines being different from pharmaceuticals, 2) evidence and effectiveness, and 3) barriers to herbal medicine use.

#### Herbal medicines being different from pharmaceuticals

The first theme identified was that herbal medicines differed from pharmaceuticals. This was the primary reason people chose herbal medicines as a treatment. For most participants the decision to use herbal medicines for the first time was the consequence of a disappointing experience with conventional medicine (not working or unwanted side effects). Some participants were initially introduced to taking herbal medicines via family members. For all participants the perceived qualities of herbal medicines was the main factor that differentiated them from pharmaceutical products. *Herbal medicines being different from pharmaceuticals* incorporated the subthemes of *herbal medicine qualities* and *appropriate use*.

*Herbal medicine qualities*. All participants discussed beliefs about the qualities of herbal medicines. They described a range of attributes herbal medicines have that differentiated them from pharmaceuticals. Herbal medicines were described as being safe, having fewer side effects than pharmaceuticals, and being gentle or healing; participants felt that these qualities were because herbal medicines are natural. Being gentler with fewer side effects appeared to be the primary reason someone would choose an herbal medicine over a pharmaceutical.

Betty: In many regards they're, I guess they're a lot more gentle, a lot more soothing than, than a lot of the Western [pharmaceutical] medicines.

Emily: Xanax also puts you in a bit of a state of, I could stand stirring a cup of coffee for 10 minutes and just be staring at the window [and] not realise I'm doing anything. Whereas Anxioton [herbal formula] is just a nice calm, you don't feel like it's mind altering at all like the Xanax ….

A common theme was the belief that being natural was an important quality of herbal medicines, and they were thus preferred over pharmaceutical medicines. Some participants believed it should be the first choice of treatment for this reason.

Linda: … if it's based on a natural process and it's not synthesised then it should always be the option before taking some unknown drug that's been made chemically. If you can treat your body, or your mental health, or your physical heath with something more natural it should always be used as a first priority.

In contrast, there were also concerns about the belief that natural is better, and this belief should not be the sole reason for making treatment decisions. Most participants had these concerns.

Kelly: I think that I don't have an idea that if it's natural it's good, and if it's not natural it's bad. I think that's a stupid, dangerous, ridiculous bifurcation … it frightens me the idea that people may be very vulnerable, not psychologically vulnerable so much as physically vulnerable, might get access to ingredients that can have an active effect on your system, without knowing really what the implications of that are.

*Appropriate use of herbal medicines*. Whether or not herbal medicines are suitable for a particular situation was an important factor in decision-making for all participants. All participants believed that both conventional and herbal medicines were important treatment options, and what they chose to use was dependent on each situation. The need to use conventional medicine in certain situations was recognized by all participants.

Greg: I think [for] a lot of lifestyle diseases and prevention things, I think that herbal medicine has a very strong role to play …. For things more serious, like you know, things requiring surgery, or you know cancer, then obviously it's a bit more limited there.

Mary: … some people say try, you know, modern [conventional] medicine first and if it doesn't work use herbal. I have no issue with using either one first, or using them both together. I think it depends on what you need it for. Yeah, totally, and it depends on the person again. What works for one may not work for someone else.

Although the qualities of herbal medicine can attract people to using them, conversely they can be a barrier to use. For example, while the gentle action of herbs (i.e., having fewer side effects) was desirable, being slower to act was considered a problem especially when a fast action might be needed to relieve symptoms or treat an infection.

Betty: … the severity of an illness … if I'm dealing with something that just needs to be knocked on the head really quickly, which herbal medicines tend to need a bit of time.

Sally: … it's probably a slower approach to addressing a problem than prescription [pharmaceutical] drugs.

#### Effectiveness and evidence

Evidence and effectiveness were widely discussed by all participants and included their own experience of herbal medicines, the experience of others (descriptive norms), and information sources. These beliefs influenced a person's decision about whether or not they would take a particular herbal medicine for a specific condition. Those who were more ambivalent about their effectiveness or who questioned the evidence for their effects seemed less likely to use herbal medicine compared to those who had stronger beliefs about effectiveness. However, all participants believed herbal medicines had some level of effectiveness and that there was evidence for this. If there was evidence (as defined by the participants) that an herb had a particular effect, then they had the expectation that it should be effective.

*Previous experience (own)*. Having had previous experience with herbal medicines was an important form of evidence for effectiveness. A previous positive experience meant that a person was more likely to try herbal medicines again in the future, even if they had had a negative experience in another context. For one participant a negative experience with herbal medicines meant that she was reluctant to try them again.

Kelly: I would have to say that I am probably a bit ambivalent. On the one hand I recognise that there's a very long tradition, a great deal of clinical experience for however many thousands of years I don't even know. On the other hand my own personal experience has not been a positive one. I've not noticed any benefit from trying herbal medicines. So I'm not really sure how I feel about, about them.

Most participants described positive experiences; however, they described the effects of herbal medicines differently to those of pharmaceuticals. For example, participants referred to herbal medicines as calming, rather than describing them as having a direct effect on anxiety symptoms. Mary discussed using valerian to “calm her down” when she is stressed, and Linda, who took St John's wort for depression, said it “calms me down cause you kind of get a bit anxious.” The two participants who had more severe anxiety had contrasting experiences.

Kelly: … I guess my initial decision to try herbal medicines was influenced by a lack of positive experience with seeing a doctor. And then my decision to not continue to try herbal medicines was influenced by the fact that I didn't perceive that it was working.

Emily: It [herbal medicine] honestly works … I was only taking two of the Anxioton [herbal formula] or something, and I didn't notice much, and she [the naturopath] said look can you just do what I ask and take the four a day. And I went oh ok, did it, and then all of a sudden it was like [I could cope without having to ring my dad to calm me down].

*Previous experience (others)*. When asked what they believed was good evidence all participants valued anecdotal evidence and considered it important when making decisions about using herbal medicines. Most participants valued this more highly than other forms of evidence, including scientific evidence. Even Kelly, who was highly educated with a good understanding of research methodology and evidence-based treatments, believed that anecdotal evidence was important when evaluating the effectiveness of herbal medicines.

Kelly: Well I think there is two sides to that. One is if people take them and report a benefit, and that happens for many here in many different situations with many different clients … I think that's good evidence.

David: Well proof of the pudding to a certain extent. That there's some evidence that it works … that someone had told me by word of mouth that they'd been taking this whatever it was for a period of time and they felt that it cured them, I'd probably be prepared to give it a go.

*Information sources*. While all participants believed there was evidence for the effectiveness of herbal medicines, there were varied opinions about what was considered good evidence. Participants had various levels of knowledge about herbal medicines that influenced their beliefs about them and how they used them. They obtained their information from a range of sources, including family and friends, the Internet, and health practitioners—some of whom were herbalists. Only one participant relied solely on a naturopath (also an herbalist) for information and prescription of herbal medicines, which contrasted with another participant who only self-prescribed herbal medicines and relied on information from friends or health food shop assistants. All other participants used a range of sources for herbal medicine information.

Greg: From hearing anecdotal stories … [and] through the naturopath herself I suppose … [when I was younger my partner was studying herbal medicine and] I was reading a lot of her notes … and just conversations with different people over the years. Oh, I subscribe to Go Health, and when I go to GoVita I just read their magazine a lot, and sometimes I'll purchase products based on some articles.

People did seek information from their doctors; however they would make their decisions about treatments after considering other information sources as well.

Emily: Everything that's happened to me ever in my life, whether it's a cold, my hands, my anxiety, I'll go to my doctor and find out what it is. But then I'll go to my naturopath or my healer and say, what have you got? … 'cause I still trust the GPs … and I think making an informed decision with both of the information [sources] that you can get [is the smart thing to do].

#### Barriers to using herbal medicines

Barriers to using herbal medicine incorporated the subthemes of cost, confusion, and treatment effectiveness (both herbal medicine and conventional medicine). All participants discussed their beliefs about the things that either empower them or prevent them from using herbal medicines.

*Cost*. For most participants the cost of herbal medicines was a concern that could be a barrier to using them. However, one participant was “not influenced by the cost of herbal medicines.” Two participants believed that the price of herbal medicines indicated their quality and consequently how effective they are.

Linda: I think what I've worked out is that the better ones obviously cost a bit more … Sometimes I can't afford it. I'll always buy that [a herbal medicine] before buying something from a chemist, ah or going to a doctor … but I'm a single mum and sometimes I actually can't afford anything.

Sally: There's probably some things out there that I wouldn't mind trying, but I think they're just overpriced … So price does come into it for certain things. But then I'll pay … I don't buy the cheaper version. I'll buy good quality things. But there's a certain cut-off point where it might put me off trying something because of the price.

*Confusion*. Most participants were confused about herbal medicine information. This confusion could relate to not knowing which product is most suitable to use and what information sources are reliable.

Linda: There's a lot of, you know, this works for me, and this works for me, and everyone's got their opinion on what works for them … you sort of listen to different people and you go to the health food shop and they tell you about something and yeah. And it's hard to sort of know what you should be buying.

One participant was confused by the different types of herbal medicine preparations and described a perceived difference in their effectiveness.

Greg: … there seems to be two different schools of naturopathy … and they [drop doses] don't seem to have as an effective response …. When I've gone to a naturopath that uses the full [therapeutic] dose it seemed to work better …. When I have used the drop dose … sometimes they still work, but not as much … and sometimes they haven't worked at all to be honest.

*Treatment effectiveness*. As mentioned above, previous experience influences beliefs about effectiveness, and it can also be a barrier to using herbal medicines when either conventional medicine is already working for someone or if herbal medicine is not working.

David: I've looked at them [herbal medicines] and thought about them, and I've sort of been a bit reluctant to try them because what I'm taking seems to be, well seems to work quite well.

Sally: … sometimes it's a reoccurring health problem, and something [a herbal medicine] might be working, but the barrier might be it's not working well enough … and you're willing to try … a more traditional [conventional] medicine approach.

## Discussion

This study aimed to explore the beliefs and attitudes towards herbal medicines of adults who have experienced anxiety. Three major themes were identified that influenced the participants’ herbal medicine use: herbal medicines being different from pharmaceuticals, evidence and effectiveness, and barriers to using herbal medicines. Previous research in other cohorts suggests that users of herbal medicines are likely to have post-modern values incorporating beliefs about the importance of natural remedies (O'Callaghan & Jordan, 2003), holism (McFadden, Hernández, & Ito, 2010), having control over their health (Thomson et al., 2014), and a dissatisfied attitude towards a medical encounter or treatment outcome (Sirois & Gick, 2002). The current study confirms that similar beliefs exist in this sample of adults experiencing anxiety.

In this study, the participants identified the qualities of herbal medicine as the primary reason for seeking an alternative treatment. The perceived differences between herbal medicines and pharmaceutical medicines appeared to draw people towards using herbal medicines. These differences are important to people as they provide an alternative choice if people are dissatisfied with conventional treatment. This finding is consistent with previous research identifying dissatisfaction with the treatment outcome to be important for users of herbal medicines in other clinical groups (Shumay et al., 2002; Sirois & Gick, 2002). In the current study, participants spoke about their dislike of side effects and potential harm from pharmaceutical treatments, which in one participant with more severe anxiety caused her anxiety to increase. These qualities of pharmaceuticals may cause people to believe they have less control over their health and consequently drive people to seek alternative treatments.

The current study found that various types of evidence were important; however anecdotal evidence was the most influential on treatment decision-making. This result is consistent with previous research on psychiatry patients (Alderman & Kiepfer, 2003) and cancer patients (Saini et al., 2011) who relied heavily on friends and family as information sources about herbal medicines. This reliance on anecdotal evidence could be related to trust. People have more trust in others they have close relationships with compared to authority figures. Rejection of authority is an attitude found to predict herbal medicine use (O'Callaghan & Jordan, 2003). Although not explored directly in this study, we did not find evidence for this attitude. All participants were willing to consult with doctors if needed. Alternatively, people who have previous experience with a specific health treatment are considered experts of their own experience by others in their community (Cotten & Gupta, 2004). This tendency may partially explain why people value anecdotal evidence over scientific evidence.

The varied experiences of effectiveness described by participants may be explained by the different ways in which herbal medicines were used and how anxiety was experienced. No two people experienced anxiety in the same way, and each person took different herbal medicines in different contexts. These unique experiences influenced their beliefs and attitudes. The two participants with more extreme anxiety symptoms had opposing experiences with their treatments. One had a very positive experience, with their prescription of herbal medicines being managed by an herbal medicine practitioner, while the other had a negative experience following over-the-counter advice from an herbalist in a health food store. The different contexts of prescription may provide some explanation for the difference in perceived effectiveness. The person seeing the practitioner was also supported in other ways including lifestyle change, nutritional supplements, and a therapeutic alliance. It is possible that this holistic approach provides a greater benefit to people compared to over-the-counter sales.

The belief that herbs are slower to act than pharmaceutical drugs was a barrier to using herbal medicines. This belief contradicts some of the research evidence for particular herbs. For example, kava has been demonstrated to have a rapid effect similar to that of benzodiazepines in relieving anxiety symptoms (Sarris, LaPorte, & Schweitzer, 2011). However, it seems there is a lack of evidence for many herbal anxiolytics, so it is difficult to determine the speed of action of many of the herbs taken. It could be that people are not taking adequate doses of these medicines or have taken poor quality products that do not provide a reliable result, particularly when self-prescribing. Future research should explore the reasons behind this belief. Despite herbal medicines being considered an affordable form of medicine (World Health Organization, 1998), most participants believed cost was a barrier to using herbal medicines and believed they were expensive. This belief is partly due to pharmaceutical medicines being subsidized in Australia, making them an affordable treatment option.

Using a qualitative approach allowed for exploration of data, which uncovered some unique findings that can be used to inform future research. However, there are limitations in this study that need consideration. This was part of a mixed-methods postgraduate study that had time and financial constraints. It is possible that other themes may have been identified if there were a greater number of participants. However, this was a special interest group with a specific scope, and the analytic purpose of the study was achieved (Mason, 2010). In consideration of these restrictions, modest claims have been made about the findings. As this is a qualitative study seeking to explore phenomena in this specific cohort, no generalizable conclusions can be made from these results.

## Conclusion

The study showed that participants tend to rely on herbal medicines because they believe they are effective and have different qualities from pharmaceutical drugs. This tendency is based on their belief that they are safe and natural, as well as on their previous experience and the experiences of family and friends. However, participants acknowledged that the available information about herbal medicines can be confusing, that herbal medicines are not able to treat all health problems, and therefore conventional medicine is also important. The study also demonstrated a reliance on non-professional information sources and anecdotes, as well as beliefs about the safety of natural treatments that expose people to the risk of taking ineffective treatments or herb–drug interactions. Further research could focus on how to balance patient autonomy and empowerment, while ensuring safe and effective treatment for anxiety. The results of this study will inform future research, and provide guidance for health practitioners.

As people use herbal medicines as an alternative to evidence-based treatments or concurrently with pharmaceutical treatments, it is critical they be provided with information about treatment options, including possible interactions with pharmaceuticals and the suitability of specific treatments for their particular needs. Therefore, future research needs to contribute to education strategies both for people experiencing anxiety who use herbal medicines and for health practitioners. It is critical that health practitioners supporting people with anxiety have knowledge of herbal medicines, so they can educate their patients about the potential problems of self-prescribing and encourage discussion about herbal medicine use. In addition, concerns about not being taken seriously were raised by participants in this study, which can push people away from healthcare providers or cause them to not disclose their medicine use. Therefore, when health practitioners have discussions with people about treatment options, they need to empathize with them and respect their beliefs and treatment decisions. This approach is particularly important for people with more severe anxiety, due to the risk of interactions with pharmaceuticals or of not receiving suitable treatment to help manage anxiety symptoms.

## Appendix A

Research Instrument: Interview Questions

Thank you for agreeing to participate in this study and volunteer your time. This interview will seek to explore what your beliefs are about herbal medicines, and to understand why and how you use herbal medicines.

1. Could you tell me how you first became aware of herbal medicine?
2. What are your beliefs about herbal medicines?
3. What were your expectations towards herbal medicine?*
4. Do you think herbal medicines are effective?
    a. Do you think there is evidence for the effects of herbal medicines?^#^
    b. What do you believe is good evidence?^#^
5. When do you think herbal medicines should be used?
6. What is your understanding of how herbal medicines work?
7. Do you have any concerns about herbal medicines?
8. What factors influence your decision to use (or not use) herbal medicines?
9. What are your main sources of information about herbal medicine?*
10. What role does the cost of herbal medicine play regarding your decision to use it?*
    a. Are there any other barriers to you using herbal medicine?^#^
11. Could you tell me about your experience with herbal medicines?
    a. What kind of benefit (or not) they have provided?^#^
12. How did you come to use herbal medicine?*
13. What have you taken herbal medicines for?
14. What herbal medicines have you taken?
15. How have you taken herbal medicines?
16. Do you tell your doctor that you use herbal medicines?*
17. Do you tell other health care providers that you use herbal medicines?
18. Is there anything else you would like to comment on in relation to the questions we have covered today?

Additional possible questions:

- Do you think of herbal teas as a medicine?

Note:

*Questions from Joos, Glassen, and Musselmann (2012).

^#^Additional probing questions may be used that are relevant to the primary question.
